# Multi-Layer Omics Analysis Identifies Anxa3 and Coro1a as Candidate Targets of Pien Tze Huang in a Mouse Model of Liver Fibrosis

**DOI:** 10.3390/biomedicines14071550

**Published:** 2026-07-10

**Authors:** Hao Wu, Longhui Gao, Xianglong Zhao, Xiangyi Li, Yunxiao Lin, Luan Chen, Lixing Li, Lu Shen, Wei Bao, Jinhang Zhu, Cong Huai, Zhiliang Chen, Yichao Zhuang, Shengying Qin

**Affiliations:** 1 Key Laboratory for the Genetics of Developmental and Neuropsychiatric Disorders (Ministry of Education), Bio-X Institutes, Shanghai Jiao Tong University, Shanghai 200030, China; 2 Fujian Provincial Key Laboratory of Pien Tze Huang Natural Medicine Research and Development, Zhangzhou Pien Tze Huang Pharmaceutical Co., Ltd., Zhangzhou 363000, China; 3 Department of Liver Surgery, Liver Cancer Institute, Zhongshan Hospital, and Key Laboratory of Carcinogenesis and Cancer Invasion (Ministry of Education), Fudan University, Shanghai 200032, China

**Keywords:** liver fibrosis, Pien Tze Huang, DNA methylation, mouse model, CCl_4_

## Abstract

**Background/Objectives:** Liver fibrosis, a wound-healing response to chronic liver injury characterized by excessive extracellular matrix (ECM) accumulation, represents a major global health burden with no approved anti-fibrotic therapies. Pien Tze Huang (PZH), an officially approved traditional Chinese medicine (NMPA Drug Approval No. Z35020243), has demonstrated hepatoprotective effects, yet its epigenetic mechanisms in fibrosis treatment remain unexplored. **Methods**: We performed the first integrated methylome–transcriptome–proteome analysis to investigate PZH’s anti-fibrotic mechanisms in a CCl_4_-induced mouse model using reduced representation bisulfite sequencing (RRBS), RNA-seq, and TMT-labeled LC-MS/MS. **Results**: We identified 10,974 differentially methylated loci (DMLs) and 773 differentially expressed genes (DEGs) modulated by PZH treatment. Integration analysis revealed ANXA3 and CORO1A as candidate therapeutic targets exhibiting significant inverse methylation-expression correlations validated at both transcriptomic and proteomic levels. Notably, PZH treatment modulated the CRLF-CLCF1 cytokine complex and the EGR-3 transcription factor network (42/44 genes enriched), suggesting broad transcriptional reprogramming in fibrotic liver. Protein–protein interaction (PPI) analysis highlighted key gene pairs such as *Dnmt1-Uhrf1*, *Cbfb-Runx1*, and *Col4a1-Col4a2*, implicating PZH in epigenetic maintenance, transcription factor regulation, and ECM remodeling. **Conclusions:** These findings suggest mechanistic insights into PZH’s multi-target anti-fibrotic effects and offer a rationale for developing potential therapeutic targets for liver fibrosis.

## 1. Introduction

Liver fibrosis is a wound-healing response to chronic liver injury, characterized by the excessive accumulation of extracellular matrix (ECM) components. It represents the final common pathway for virtually all chronic liver diseases [1]. Despite advances in understanding the molecular mechanisms, no effective anti-fibrotic treatments are currently available for clinical use [2]. Therefore, elucidating the molecular mechanisms underlying liver fibrosis remains essential for developing targeted therapies.

The pathogenesis of liver fibrosis involves various cellular and molecular mechanisms, including the activation of hepatic stellate cells (HSCs), chronic inflammation, oxidative stress, and epigenetic changes such as DNA methylation [3]. Recent studies have highlighted the role of DNA methylation in the pathogenesis of liver fibrosis. DNA methylation patterns are profoundly altered, exhibiting a bidirectional pattern characterized by global genomic hypomethylation concurrent with promoter-specific hypermethylation at key fibrosis-related genes [4,5]. This epigenetic imbalance contributes to the dysregulation of genes controlling HSC activation, inflammatory responses, and ECM production.

DNA methylation refers to the addition of a methyl group to the 5′ position of cytosine residues in CpG dinucleotides, which is generally associated with transcriptional repression [6]. This epigenetic modification regulates genes involved in essential cellular processes, including cell cycle control, apoptosis, and detoxification, which are implicated in the activation of HSCs and the development of liver fibrosis [7,8].

Aberrant DNA methylation has been increasingly implicated in liver fibrosis [9]. Global hypomethylation of pro-fibrotic genes (e.g., *COL4A1/2* and *SPP1*) and promoter hypermethylation of regulatory genes contribute to hepatic stellate cell activation and extracellular matrix accumulation [3,10]. For example, hypermethylation of *RCAN1* and *PSTPIP2* has been associated with impaired cellular functions and fibrotic progression [11,12]. DNA methyltransferase (DNMT)-mediated promoter CpG methylation contributes to transcriptional silencing of antifibrotic genes during liver fibrosis progression [5]. Importantly, pharmacological inhibition of DNA methylation agents, such as 5-aza-2′-deoxycytidine (5-azadC), has been shown to inhibit hepatic stellate cell (HSC) activation and proliferation [13], suggesting epigenetic regulation as a potential therapeutic target [5].

Pien Tze Huang (PZH), a renowned traditional Chinese medicine prescription originating from the Ming Dynasty, has been officially approved by China’s National Medical Products Administration (NMPA) for clinical use (Drug Approval No. Z35020243). PZH has been widely used for centuries in liver-related diseases, and has shown hepatoprotective effects against in experimental modes, including carbon tetrachloride (CCl_4_)-induced fibrosis, alcohol-induced liver injury, and high-fat diet-induced NAFLD [14,15]. Mechanistically, PZH has been reported to modulate multiple intracellular signaling pathways, including the TGF-beta 1/Smad pathway, which is closely associated with HSC activation and fibrogenesis [14,16]. Among its constituents, ginsenoside Rg1 has been reported to regulate processes in liver diseases. Given its multi-component nature, PZH may act as a potential modulator of epigenetic regulation, although its effects on DNA methylation machinery and liver fibrosis remain unclear.

Effective anti-fibrotic interventions may restore epigenetic homeostasis by correcting aberrant DNA methylation patterns, including both hypomethylation and hypermethylation patterns in fibrotic liver. Agents that modulate DNMT activity or methylation metabolism may normalize the methylation landscape, leading to reactivation of silenced anti-fibrotic genes and suppression of profibrotic genes. Based on these considerations, we hypothesized that PZH may exert therapeutic effects in liver fibrosis through modulation of DNA methylation.

This study addresses this critical knowledge gap by employing integrated methylome, transcriptome, and proteome analysis to characterize PZH’s epigenetic mechanisms in liver fibrosis treatment. We hypothesize that PZH may exert its therapeutic effects by modulating DNA methylation patterns, thereby influencing gene expression and cellular pathways associated with liver fibrosis. The results could provide new insights into the molecular mechanisms of PZH and contribute to the development of novel therapeutic strategies for liver fibrosis.

## 2. Materials and Methods

### 2.1. Pien Tze Huang and Reagents Description

Pien Tze Huang (PZH) is a complex traditional Chinese medicine formulation officially approved by the NMPA (Drug Approval No. Z35020243). PZH comprises multiple botanical and animal-derived ingredients, the primary active constituent groups of which include bile acid derivatives, saponins, triterpenoid compounds, and other bioactive botanical extracts. The batch-to-batch consistency of PZH is ensured through standardized manufacturing protocols governed by NMPA quality control regulations. Quality control measures include testing for heavy metals, pesticide residues, and microbial contamination. PZH was obtained from and authenticated by Zhangzhou Pien Tze Huang Pharmaceutical Co., Ltd. (Zhangzhou, China), and a single batch was used throughout the study. PZH powder was dissolved in distilled water and diluted to a concentration of 25 mg/mL. CCl_4_ was dissolved in 10% corn oil at a volume ratio of 1:1.

### 2.2. Animals and Experimental Design

Forty-five 6-week-old male C57BL/6 mice were obtained from Shanghai Jieshijie Laboratory Animal Co., Ltd. (Approval number: SCXK-Shanghai-2018-0004). All animal experiments were conducted in accordance with the guidelines of the National Institutes of Health of China for the care and use of laboratory animals. These experiments were approved by SRCMO IACUC (Shanghai, China; approval no. 2017-0033, 11 September 2017).

Animals were randomly allocated to experimental groups using computer-generated randomization. Treatment allocation was concealed from investigators administering treatments and assessing outcomes.

Histological analyses were performed under blinded conditions, and raw omics data were processed with group identities concealed using standardized bioinformatics pipelines. Mice were randomly assigned to three groups (*n* = 15 per group): (1) Vehicle group: intraperitoneal injection of 10 µL/g corn oil twice weekly plus intragastric distilled water once daily; (2) CCl_4_ group: intraperitoneal injection of 10 µL/g 10% CCl_4_ twice weekly plus intragastric distilled water once daily; (3) PZH group: intraperitoneal injection of 10 µL/g 10% CCl_4_ twice weekly plus intragastric PZH (0.25 mg/g) once daily. After 12 weeks, all mice were sacrificed, and livers were harvested for RRBS, RNA-seq, and TMT-labeled LC-MS/MS.

### 2.3. Sirius Red Staining and Quantification

Liver tissues were fixed in 4% paraformaldehyde, paraffin-embedded, and sectioned at 5 μm thickness. Sections were stained with Sirius Red (0.1% Direct Red 80 in saturated picric acid) for 1 h at room temperature, followed by washes in acidified water and dehydration through graded ethanol. Collagen deposition was visualized under polarized light microscopy. The Sirius Red-positive area relative to total hepatic parenchyma was quantified using image analysis software. Statistical significance was assessed by one-way ANOVA followed by Tukey’s post hoc test.

### 2.4. Reduced Representation Bisulfite Sequencing and Data Analysis

Genomic DNA was extracted using the QIAamp DNA Mini Kit (Qiagen, Hilden, Germany). RRBS libraries were prepared as follows: DNA was digested with MspI, followed by end-repair, A-tailing, ligation of methylated adapters, and gel purification (40–220 bp). Bisulfite conversion was performed using the EZ DNA Methylation-Gold Kit (Zymo Research, Irvine, CA, USA), followed by PCR amplification. Libraries were quantified by Qubit 2.0, assessed on an Agilent 2100 Bioanalyzer (Agilent Technologies, Santa Clara, CA, USA), and sequenced on the Illumina MiSeq platform (Illumina, San Diego, CA, USA).

General quality control was conducted using FastQC v0.11.9. Reads of low quality (error ratio > 0.01, read length < 70 bp, or unpaired reads) and adaptors were eliminated using Trim Galore v0.4.1 [17]. The filtered datasets were aligned to the reference genome mm10 using Bismark v0.15.0 [18]. Differentially methylated loci (DML) were identified using the R package DSS v2.34.0 [19]. Loci with a threshold *p*-value < 0.05 (adjusted using the Benjamini–Hochberg false discovery rate correction) and a mean methylation difference > 0.1 were considered significantly differentially methylated. The genomic context of the identified DMLs with respect to known genes and gene features was analyzed using the R package annotatr v1.21.1 [20] and ChIPseeker v1.22.1 [21].

### 2.5. RNA-Seq and Data Analysis

Total RNA was extracted utilizing the RNeasy Mini Kit (Qiagen, Hilden, Germany), strictly adhering to the manufacturer’s guidelines. The integrity of the RNA was assessed by determining the RNA Integrity Number (RIN) using an Agilent Bioanalyzer 2100. Only RNA samples exhibiting a RIN of ≥7.0 were deemed suitable for sequencing. Libraries were constructed using the VAHTS Total RNA-seq Library PrepKit for Illumina (Vazyme Biotech Co., Ltd., Nanjing, China) and sequenced on the Illumina NovaSeq 6000 platform (Illumina, San Diego, CA, USA). Sequencing depth was ≥20 million paired-end reads per sample, and raw reads were quality-controlled (Q30 ≥ 85%) and aligned to the reference genome (GRCm38) using Hisat2 [22] with a mapping efficiency ≥ 65%. StringTie v2.1.5 [23] was used to obtain the gene counts and EdgeR v3.28.1 [24] was employed to normalize the count data by TMM and identify differentially expressed genes, with a threshold |log2FC| > 1 and an adjusted *p*-value < 0.05. Genes meeting these criteria were deemed significantly differentially expressed.

### 2.6. Proteome Analysis

Samples were homogenized in SDT buffer (4% SDS, 100 mM Tris/HCl, pH 7.6, 0.1 M DTT). Proteins were quantified by the BCA assay (Thermo Fisher Scientific, Waltham, MA, USA). For each sample, 200 μg protein was digested with trypsin using the FASP method, desalted with a C18 cartridge, and lyophilized. Peptides (100 μg per sample) were labeled using the Thermo Fisher TMT kit (Thermo Fisher Scientific, Waltham, MA, USA).

Labeled peptides were pooled and fractionated by SCX chromatography on an AKTA Purifier 100 (GE Healthcare, Marlborough, MA, USA) with a KCl gradient (0–500 mM in 25% ACN, pH 3.0). Fractions were desalted and analyzed by nano-LC-MS/MS on a QE HFX mass spectrometer (Thermo Fisher Scientific, Waltham, MA, USA). Raw data were searched against the reference database using Proteome Discoverer v1.4. Proteins with fold change > 1.5 for upregulation or downregulation were considered differentially expressed proteins (DEPs).

### 2.7. Integration of DNA Methylation and Gene Expression

To clarify the relationship between DNA methylation patterns and gene expression levels, we conducted a comprehensive analysis integrating both DNA methylation and gene expression data. The initial step involved identifying genes with substantial methylation changes in their promoter regions, using criteria of a mean methylation difference > 0.1 and an adjusted *p*-value < 0.05. We then selected genes that showed significant differences in expression, with criteria of |log2FC| > 1 and an adjusted *p*-value < 0.05. Methylation and expression data were integrated using the following approach: (1) promoter regions were defined as ±2 kb from the transcription start site (TSS), based on the UCSC RefSeq mouse annotation database (GRCm38); (2) for each gene, promoter-associated DMLs that demonstrated negative correlation with expression levels (e.g., hypermethylated versus down-regulated or hypomethylated versus up-regulated) were prioritized for further exploration; (3) genes with conflicting promoter methylation signals were excluded, except for Runx1, which contained one conflicting locus among 26 total loci (Supplementary Table S4). Accordingly, all genes showing inverse correlations between promoter methylation and gene expression were retained.

### 2.8. Functional Enrichment and PPI Network Analysis

GO and KEGG enrichment analyses were performed on genes that were both differentially methylated and differentially expressed using Metascape v3.5 [25] and clusterProfiler v4.2.2 [26]. Terms with adjusted *p* < 0.05 were considered significant. Integrated gene sets were further analyzed using g:Profiler e109_eg56_p17_c1b4cc6c [27] for functional annotation. Protein–protein interaction (PPI) networks were constructed using the STRING database v11.5 [28], focusing on genes showing inverse methylation–expression relationships to identify functional modules and pathway-level interactions.

## 3. Results

### 3.1. Histological Verification of Fibrosis Induction and Treatment Response

To verify that CCl_4_ treatment successfully induced liver fibrosis and confirm the anti-fibrotic effect of PZH, we performed Sirius Red collagen staining on liver tissue sections from all experimental groups. Staining demonstrated that CCl_4_ treatment significantly increased collagen deposition compared to the vehicle control group (*p* < 0.001), confirming successful induction of liver fibrosis (Supplementary Figure S1A). PZH treatment significantly reduced collagen accumulation compared to the CCl_4_-only group (*p* < 0.001), confirming the anti-fibrotic efficacy of PZH in this model (Supplementary Figure S1B). These histological findings provide independent validation of the fibrosis induction protocol and therapeutic response to PZH, supporting the biological interpretation of the subsequent multi-omics analyses.

### 3.2. DNA Methylation Analysis

RRBS was conducted to investigate the global DNA methylation alterations in a CCl_4_-induced mouse model of liver fibrosis, with and without PZH treatment. Following filtration and quality control, the clean reads ratio ranged between 85.24% and 88.29% (Supplementary Table S1). Bismark was employed to align the reads to the mouse genome and to determine the cytosine methylation levels at a single base-pair resolution, with a unique mapping ratio ranging from 65.3% to 71.1% (Supplementary Table S1).

Utilizing the callDML function from the R package DSS, differentially methylated loci (DML) were identified from the BAM files. The analysis revealed that 10,974 CpG sites exhibited differential methylation between the PZH and CCl_4_ groups, with 4236 CpG sites showing hypermethylation and 6738 CpG sites showing hypomethylation (*p* < 0.05) (Supplementary Table S2). Among these, 1106 hypermethylated CpG sites were located within the promoter regions of 490 genes, and 1754 hypomethylated CpG sites were found within the promoter regions of 656 genes (*p* < 0.05) (Supplementary Table S2). Subsequent analyses focused on genes containing DML within their promoter regions.

**Figure 1:**
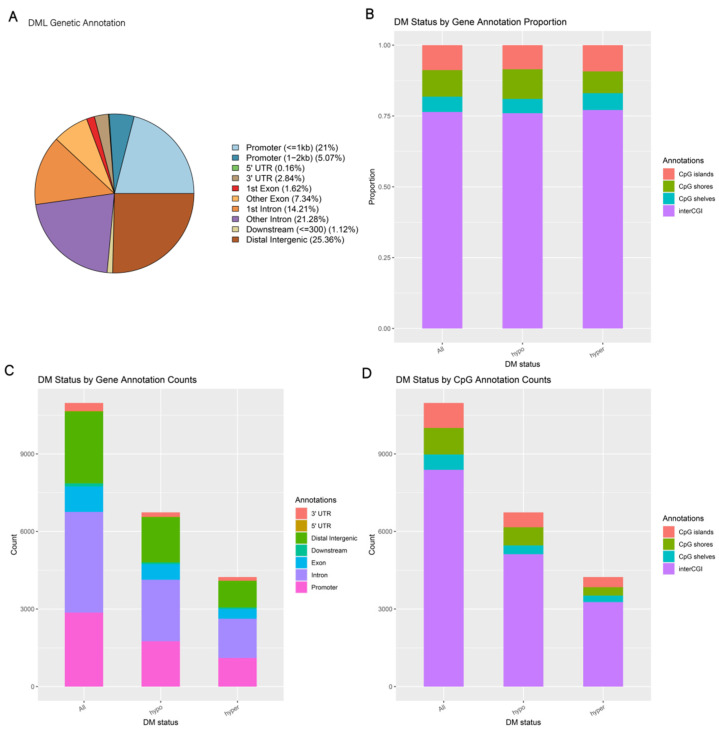
Genome-wide distribution of differentially methylated loci (DMLs) between PZH-treated and CCl_4_-treated groups. (**A**) The proportion of DMLs that overlap with 5′ UTR, exons, 3′ UTR, promoter, intron, and intergenic regions. The majority of DMLs were located in intronic regions (35.49%), followed by intergenic (25.36%) and promoter regions (26.07%), reflecting the genome-wide distribution of CpG sites in the mouse genome. (**B**) The proportion of DML with respect to CpG islands versus CpG shores, shelves, and interCGI regions. Hypermethylated DMLs showed preferential enrichment in promoter-associated CGIs compared to hypomethylated DMLs, suggesting a functional bias toward transcriptional regulation. (**C**) The distribution of hyper- and hypomethylated regions with respect to their genetic location to genes. (**D**) The distribution of hyper- and hypomethylated regions with respect to their distance to CGIs. CGI, CpG island.

The genomic context and CpG island (CGI) distribution of the DML were further examined. The results indicated that DMLss were predominantly located in intronic regions (35.49%), followed by intergenic regions (25.36%) and promoters (26.07%). A smaller proportion of DML was observed in exons (8.96%), 3′ UTRs (2.84%), and 5′ UTRs (0.16%) (Figure 1A).

Consistent with the known distribution of CpG sites in the mouse genome, the majority of DMLs were intronic. Although intronic and intergenic DMLs may also harbor regulatory potential, subsequent analyses focused on promoter-associated DMLs, as promoter regions are the most functionally characterized regulatory elements with respect to methylation-mediated gene regulation, providing the most direct and interpretable basis for integration with transcriptomic data. The regulatory potential of intronic and intergenic DMLs will be addressed in future studies as a limitation of the current work.

### 3.3. Transcriptome Analysis

**Figure 2:**
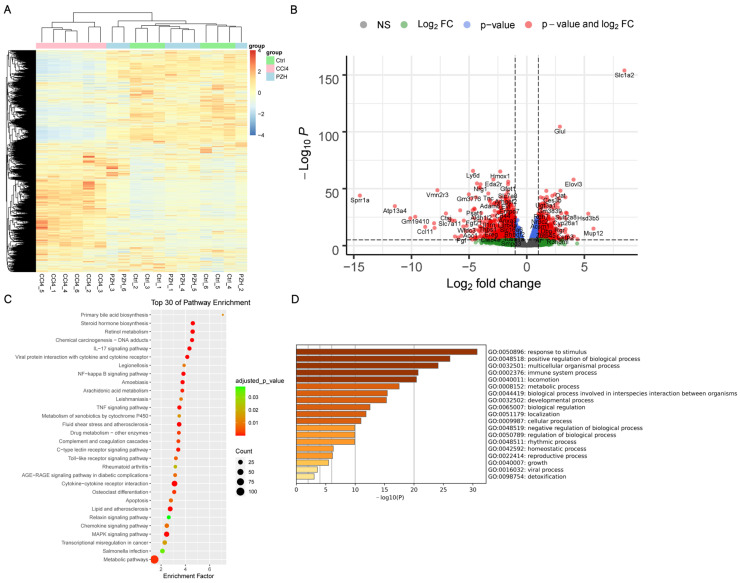
Transcriptome analysis comparing the PZH-treated group vs. the CCL_4_-treated group. (**A**) Hierarchical clustering heatmap of TMM value of all genes (TMM-normalized expression values). The row represents different genes, and the column represents different samples from three groups. (**B**) Volcano plot of DEG. The vertical axis shows −log10 (q-value), and the horizontal axis shows log_2_FC. Red and blue dots represent upregulated and downregulated genes, respectively. (**C**) KEGG pathway enrichment of DEG. The top 30 KEGG pathways are shown in the vertical axis, and the horizontal axis shows the enrichment factor. The IL-17, NF-κB, and TNF signaling pathways were the most significantly enriched, consistent with known inflammatory drivers of hepatic stellate cell activation. (**D**) GO functional category analysis of DEG. The vertical axis shows −log10 (q-value), and the horizontal axis shows GO biological process terms. FC, fold change; GO, gene ontology.

RNA-seq analysis was conducted to investigate the transcriptomic alterations between the control group, the CCl_4_ group, and the PZH treatment group. Unsupervised hierarchical clustering analysis revealed two clusters: one for the CCl_4_ group and another for the PZH group, which exhibited transcriptomic signatures closely resembling those of the control group (Figure 2A). A total of 773 genes displayed significant differential expressions between the PZH and CCl_4_ groups, with 224 genes up-regulated and 549 genes down-regulated (|log2 Fold Change| > 1, adjusted *p*-value < 0.05) (Figure 2B and Supplementary Table S3).

To uncover the potential functions of the genes affected by PZH treatment, GO and KEGG enrichment analyses were performed. The GO enrichment analysis indicated that the response to stimulus was the most significantly enriched term within the biological process category (Figure 2D). Furthermore, KEGG pathway analysis identified the IL-17, NF-kappa B, and TNF signaling pathways as the most significantly enriched pathways associated with PZH treatment in CCl_4_-induced liver fibrosis (Figure 2D). The enrichment of these pathways, particularly IL-17, TNF, and NF-κB signaling, is consistent with known inflammatory drivers of hepatic stellate cell activation during fibrogenesis.

### 3.4. Validation of Candidate Genes Involved in Epigenetic Regulation of Liver Fibrosis

**Figure 3:**
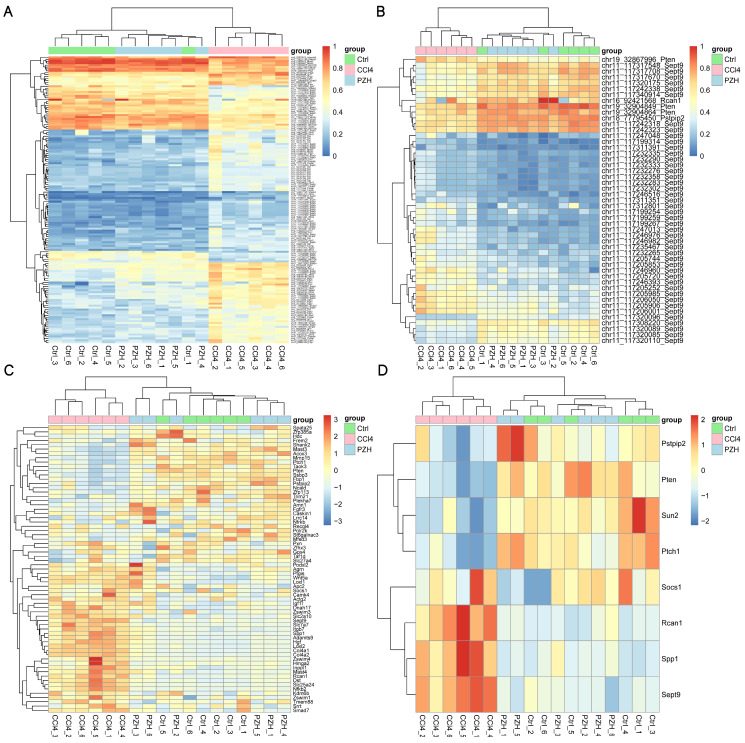
Methylation and expression patterns of previously reported fibrosis-related candidate genes. Hierarchical clustering of DMLs (**A**,**B**) and gene expression (**C**,**D**) across vehicle control, CCl_4_-only, and PZH-treated groups. PZH treatment shifted both methylation and expression patterns of these candidate genes toward those of the vehicle control group (**A**,**C**), particularly for key fibrosis-associated genes (**B**,**D**), indicating epigenetic and transcriptional normalization by PZH.

Previous research demonstrated that DNA methylation plays an important epigenetic role in the regulation of liver fibrosis. The present study summarized these previously reported genes associated with DNA methylation and corresponding expression alterations in liver fibrosis cases or animal models, compared to vehicle groups (Table 1). Unsupervised hierarchical clustering on these candidate genes related to liver fibrosis was conducted. The results identify two clusters of the CCl_4_ group and the PZH group. PZH group shared similar signatures with the control group (Figure 3A–D) in both methylation and mRNA level, which are consistent with the global transcriptome pattern (Figure 2A).

### 3.5. Integration Analysis of DNA Methylation and Expression

DNA methylation is intricately linked to gene transcription, with hypermethylation typically correlating with reduced gene expression and hypomethylation with increased gene expression, particularly when these methylation events occur in promoter regions. To further investigate the role of DNA methylation in regulating gene expression, a Venn diagram was utilized to identify 27 genes that exhibited significantly hypermethylated Differentially Methylated Loci (DML) in their promoters, which corresponded to a significant downregulation in mRNA expression (diffMethy > 0.1, log_2_FC < −1, *p*< 0.05). This group included two genes with multiple DMLs that displayed inconsistent methylation patterns. Conversely, 17 genes showed hypomethylated DML in their promoters, which were associated with upregulated mRNA expression (diffMethy < 0.1, log_2_FC > 1, *p*< 0.05) (Supplementary Tables S4 and S5).

Further analysis correlated the above genes with differentially expressed proteins. Among 64 down-regulated and 15 up-regulated proteins, Anxa3 and Coro1a exhibit positive correlations between their differentially expressed protein and mRNA levels (log_2_FC of mRNA > 1 and FC of protein > 1.5 or log_2_FC of mRNA < 1 and FC of protein < −0.67, *p* < 0.05) (Supplementary Table S6).

### 3.6. Functional Enrichment Analysis of Inversely Correlated Genes

**Figure 4:**
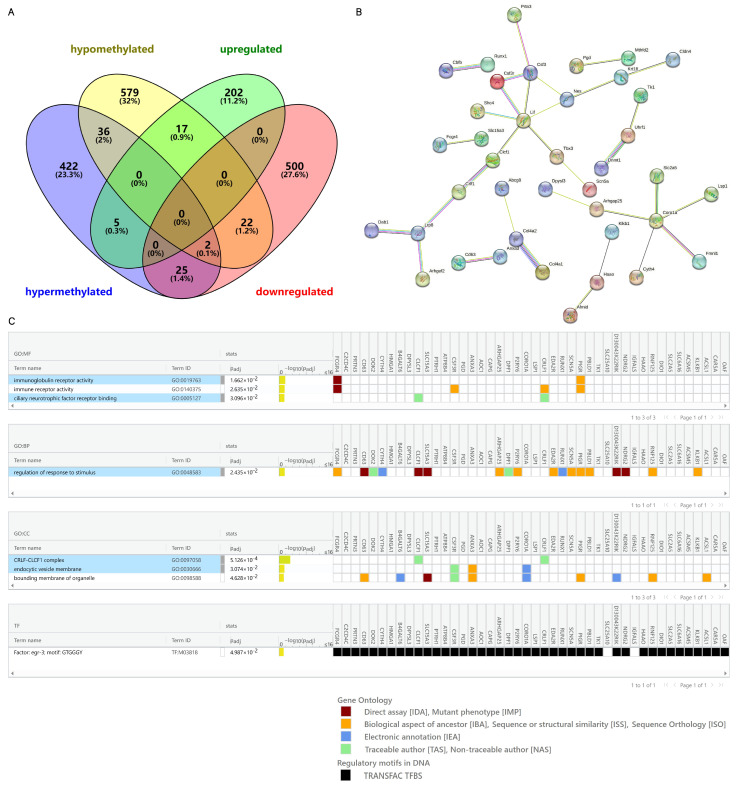
Integrated analysis of DNA methylation and mRNA expression. (**A**) Venn diagram of genes correlated to hyper- or hypomethylated DML and corresponding up- or down-regulated expression, respectively. (**B**) PPI network constructed from overlapped significantly differentially methylated and expressed genes in the PZH group. Nodes represent genes and edges represent interactions. (**C**) GO functional category and transcription factor analysis of inversely correlated genes. Columns on the right show genes enriched in each term.

Functional enrichment analysis was conducted to uncover the biological functions associated with DNA methylation and the inversely correlated gene expression between the PZH group and the CCl_4_ group. The gene ontology (GO) enrichment analysis revealed that the regulation of response to stimuli is the most prevalent term within the biological process category. In the cellular component category, the top terms were identified as the CRLF-CLCF1 complex, endocytic vesicle membrane, and the bounding membrane of organelles. Within the molecular function category, the highest-ranking terms were immunoglobulin receptor activity, immune receptor activity, and ciliary neurotrophic factor receptor binding (as depicted in Figure 4C). Additionally, the transcription factor analysis indicated that *Egr-3* was significantly enriched, being present among 42 out of the 44 genes under investigation.

### 3.7. Ppi Network of Differentially Expressed Genes

To uncover the potential relationships among the 71 differentially methylated and expressed genes, characterized by a methylation difference > 0.1, a |log2FC| > 1, and a *p* < 0.05, a protein–protein interaction network was constructed (as shown in Figure 4B). Within this network, the genes with the highest combined scores were identified as *Dnmt1-Uhrf1*, *Cbfb-Runx1*, *Col4a1-Col4a2*, *Csf3-Csf3r*, *Dab1-Lrp8,* and *Clcf1-Crlf1*, highlighting their significance in the interaction network detailed in the Supplementary Table S7.

## 4. Discussion

The present study provides a comprehensive analysis of the effects of PZH in CCl_4_-induced liver fibrosis in mice, with a particular focus on the alterations in DNA methylation and gene expression. PZH has been previously reported to ameliorate liver fibrosis through multiple pathways, including NF-κB suppression and HSC apoptosis [14,15]. Our findings of PZH-mediated epigenetic modulation complement these observations, suggesting a multi-target therapeutic approach. Zheng et al. [14] demonstrated that PZH suppresses NF-κB signaling and promotes HSC apoptosis in CCl_4_-induced fibrosis. This suggests that PZH’s anti-fibrotic effects may involve coordinated inhibition of pro-inflammatory signaling through both direct pathway modulation and epigenetic reprogramming. Similarly, Zhang et al. [15] reported that PZH inhibits HSC autophagy via the TGF-β1/Smad2 pathway. In our study, the pathway enrichment results provide biological context as follows: (1) cytokine signaling pathways (including the CRLF-CLCF1 complex and related pathways) are consistent with the known role of cytokine-mediated HSC activation in fibrosis progression; (2) ECM remodeling pathways reflect the central role of excessive ECM deposition in fibrosis, and PZH modulates CORO1A, a protein involved in cytoskeletal dynamics and autophagy regulation; (3) transcription factor networks (including EGR-3) mediate broad transcriptional reprogramming in fibrotic liver; (4) NF-κB pathway identified in KEGG enrichment involved in inflammatory responses. These pathway-level findings are consistent with previously reported anti-inflammatory and hepatoprotective activities of PZH, providing mechanistic support for the current multi-omics findings.

The results from RRBS, RNA-seq, and proteome assay revealed ANXA3 and CORO1A as key targets exhibiting significant inverse correlations between promoter methylation and expression levels. In CCl_4_-treated mice, ANXA3 and CORO1A were upregulated at both mRNA and protein levels, consistent with their pro-fibrotic roles in activated HSCs. PZH treatment restored ANXA3 and CORO1A to control levels, suggesting reversal of HSC activation through epigenetic modulation. ANXA3 has been reported to promote fibrogenesis by activating the PI3K/Akt pathway, while CORO1A dysregulation impairs autophagic flux, intensifying Endoplasmic Reticulum Stress (ERS) and inflammation. ANXA3 expression is upregulated in activated HSCs, the key effector cells in fibrogenesis. It enhances HSC activation by activating the PI3K/Akt signaling pathway, leading to increased collagen synthesis and extracellular matrix (ECM) deposition. Aggravation of Endoplasmic Reticulum Stress, inflammation, and impaired autophagy due to CORO1A upregulation intensifies ERS and promotes the production of pro-inflammatory cytokines (e.g., MCP-1, TNF-α, IL-1β). This chronic stress and inflammation drive hepatocyte injury, which activates hepatic stellate cells.

The correlation analysis between DNA methylation and mRNA expression identified 71 genes that are significantly associated with both DNA methylation and expression. These genes are involved in the regulation of response to stimulus, the CRLF-CLCF1 complex, the endosome membrane, and the boundary membrane of organelles. Among these genes, the transcription factor Egr-3 was found to be significantly enriched, which is consistent with previous studies indicating the important role of *Egr-3* in liver fibrosis [38]. Interestingly, 42 out of 44 genes were enriched in the only transcription factor EGR-3, suggesting EGR-3 could play a crucial role in PZH treatment of CCl_4_-induced liver fibrosis in mice.

PPI network analysis identified candidate hub genes, including DNMT1-UHRF1, as nodes with high connectivity within the interaction network. The gene pairs identified (e.g., DNMT1-UHRF1 in epigenetic maintenance; COL4A1-COL4A2 in ECM remodeling) are consistent with known functional partnerships in the literature, but the functional significance of these interactions in the context of PZH’s anti-fibrotic activity requires experimental validation. These findings may represent candidate targets requiring further validation in functional studies. DNA methylation, primarily occurring at the cytosine residues of CpG dinucleotides, is a crucial epigenetic modification in mammals and is catalyzed by DNA methyltransferases (DNMTs). DNMT1, the most abundant DNMT in mammalian cells, is primarily responsible for maintaining methylation patterns during DNA replication1. UHRF1 (Ubiquitin-like with PHD and RING Finger domains 1) is an essential factor for DNA methylation maintenance, as it recruits DNMT1 to hemi-methylated DNA during replication [39]. Notably, the DNMT1-UHRF1 interaction represents a potential target for PZH’s epigenetic effects, as several natural compounds have been shown to modulate this complex. The CBFB (core-binding factor subunit beta) subunit and RUNX (Runt-related transcription factor) subunit form a heterodimeric transcription factor that enhances the stability of the RUNX1 protein. Runx1 has been reported to promote liver fibrosis progression through the TGF-beta pathway [40]. Collagen type IV alpha 1 chain (COL4A1) and collagen type IV alpha 2 chain (COL4A2) are two major components of type IV collagen, which is a primary constituent of basement membranes. Aberrant expression of COL4A1/2 is associated with HSC activation and ECM accumulation in liver fibrosis [41]. Granulocyte colony-stimulating factor (G-CSF, also known as CSF3) is a cytokine that stimulates the production and function of neutrophils via its receptor, G-CSF receptor (G-CSFR, also known as CSF3R). Previous studies have suggested a role for G-CSF in liver regeneration and the amelioration of liver fibrosis [42]. Cardiotrophin-like cytokine factor 1 (CLCF1) and cytokine receptor-like factor 1 (CRLF1) form a complex that acts as a cytokine and plays a role in the regulation of immune responses and cell survival [43]. Intriguingly, the CLCF1-CRLF1 cytokine complex, which shares structural similarities with the G-CSF system, was also identified in our PPI network. Stefanovic et al. [43] reported that CRLF1 promotes HSC activation and liver fibrosis, suggesting that PZH may exert anti-fibrotic effects by modulating both G-CSF and CRLF1 signaling pathways. The Disabled-1 (DAB1) gene encodes an adaptor protein that plays a crucial role in intracellular signaling pathways. Low-density lipoprotein receptor-related protein 8 (LRP8), also known as APOER2, is a member of the low-density lipoprotein receptor family. Previous studies have suggested a role for DAB1 and LRP8 in the regulation of cellular processes such as migration and proliferation [44].

Based on the integrated multi-omics findings of this study, we propose a mechanistic concept for PZH’s anti-fibrotic activity as follows: PZH, through its multi-component composition (including bile acid derivatives, saponins, and botanical bioactives), modulates DNA methylation patterns and gene expression programs in the fibrotic liver, leading to: (1) normalization of the methylation-excision equilibrium via modulation of DNMT-UHRF1 axis components (as suggested by the DNMT1-UHRF1 interaction network finding); (2) suppression of inflammatory cytokine signaling (as supported by CRLF-CLCF1 complex modulation); (3) transcriptional reprogramming of the EGR-3 network and related transcription factors; and (4) downregulation of ECM production pathways (consistent with the known anti-fibrotic activity of PZH as documented in the literature). The identified targets ANXA3 and CORO1A represent plausible downstream effectors of these multi-level perturbations, linking PZH’s epigenetic modulation to specific effector proteins. Nevertheless, identifying which individual PZH constituents mediate each effect remains a limitation and a priority for future pharmacological dissection.

It is important to acknowledge that the observed DNA methylation changes following PZH treatment may represent either primary epigenetic effects on the hepatic epigenetic machinery or secondary consequences of the reduced inflammation and fibrosis resulting from PZH’s pharmacological activity or a combination of both. In vivo studies such as this one cannot definitively distinguish between these possibilities, as methylation changes in tissue-level analyses reflect the net result of cellular processes, including direct epigenetic modulation and indirect effects secondary to phenotypic changes. Future in vitro studies using isolated primary hepatocytes or hepatic stellate cells treated directly with PZH or individual PZH constituents could help differentiate primary from secondary epigenetic effects by isolating cell-type-specific responses free from the confounding influence of whole-organism physiology.

The current study has several limitations. (1) Sample size: the number of biological replicates per group for each omics platform (*n* = 6 per group) provides adequate statistical power for detecting moderate-to-large effect sizes but may be underpowered to detect smaller changes or rare events. (2) Biological variability: individual animal responses to CCl_4_-induced fibrosis and PZH treatment show inherent biological variability, which may affect the robustness of some identified targets. (3) Single time-point design: this study captures a snapshot of fibrosis progression and treatment response at a single experimental endpoint, limiting the ability to assess temporal dynamics of methylation, transcriptional, and proteomic changes. (4) In vivo design: while the CCl_4_ mouse model recapitulates key features of human liver fibrosis, it cannot fully replicate the complexity of human chronic liver disease. (5) Causality: the observed correlations between methylation and expression changes do not establish direct causal epigenetic regulation, and functional validation experiments are required to confirm the mechanistic role of identified targets. (6) Due to sample availability, omics results lacked additional validation.

In conclusion, this study demonstrates that PZH treatment modulates DNA methylation and gene expression programs in a CCl_4_-induced mouse model of liver fibrosis. ANXA3 and CORO1A are identified as candidate therapeutic targets whose methylation and expression changes are inversely correlated across methylome, transcriptome, and proteome datasets. The EGR-3 transcription factor network and related signaling pathways represent candidate mechanisms that may contribute to PZH’s anti-fibrotic activity. These findings provide a rationale for developing ANXA3, CORO1A, and related pathways as potential therapeutic targets for liver fibrosis, pending further functional validation. Future studies should focus on independent replication in larger cohorts, cell-type-specific validation, and functional assays (e.g., siRNA knockdown, CRISPR activation/inactivation) to establish causality between identified targets and fibrotic phenotypes.

## Figures and Tables

**Table 1:** Candidate genes involved in epigenetic mechanisms in liver fibrosis.

Hypomethylated Genes in Liver Fibrosis Group	Hypermethylated Genes in Liver Fibrosis Group	Reference
*Spp1*		[3]
	*Rcan1*	[11]
*Apc2* *Wnt5a* *Actg2* *Loxl1* *Loxl2* *Col4a1/2*	*Adamts9* *Mmp15* *Smad7* *Pten* *Hgf*	[10]
*Zfp113* *Kl* *Igf1r*	*Zswim3* *Amn1* *Plekha7* *Slc25a24* *Frem2* *Zfp385a* *Mast3* *Mast4* *Mast5* *Mast6* *Efcab3* *Nfrkb* *Podxl2* *Taok3* *Inppl1* *Hmga2* *Zswim1* *Zswim2* *Zswim3* *Zswim4* *Spata25* *Ptgis* *Pstpip2*	[12]
*Ssbp3* *Rik* *Taf1d* *Rik* *Itgb7* *Slc27a4* *Lrrc14* *Recql4* *Polr2k* *Pxn* *Acox3* *Rik* *Pisd-ps3* *Trim21* *Hdc* *Slc7a7*	*Erdr1* *Rn4.5s* *Dnah17* *Rik* *Zfhx3* *Mir3108* *Caskin1* *Dst* *Add2* *Mfsd3* *Recql4* *Srrt* *Ncald* *Mir3470b* *Shank2* *Slc2a10* *Sun2* *Sept9* *St6galnac3* *Cyp24a1* *Wnt3* *Tmem88* *Kdm6b* *Agrn*	[29]
	*pten*	[30]
	*sept9*	[31]
	*socs1*	[32]
	*Ptch1*	[33]
	*Pten*	[34]
	*camk4* *fzd10* *prkcb* *tcfeb* *fgfr3* *gpx4* *hoxd3* *nfkb2*	[35]
	*fbp1*	[36]
	*Ptch1*	[37]

## Data Availability

The datasets generated during the current study are available in the NGDC Genome Sequence Archive (ID: CRA036419).

## Supplementary Materials

The following supporting information can be downloaded at https://www.mdpi.com/article/10.3390/biomedicines14071550/s1: Figure S1. Sirius Red staining and quantification. Table S1: QC and mapping statistics of RRBS. Table S2: Differentially methylated loci between the PZH and CCl4 group. Table S3: Differentially expressed genes between the PZH and CCl4 group. Table S4: DML located in the promoter of genes inversely correlated with DEG. Table S5: DEG inversely correlated with DML located in promoters. Table S6: DEG identified by proteome analysis. Table S7: PPI statistics.
