# Supplementary figures and images for: Multi-Layer Omics Analysis Identifies Anxa3 and Coro1a as Candidate Targets of Pien Tze Huang in a Mouse Model of Liver Fibrosis
Source: Biomedicines. 2026 Jul 10;14(7):1550. doi: 10.3390/biomedicines14071550 (PMC13406053; doi:10.3390/biomedicines14071550)

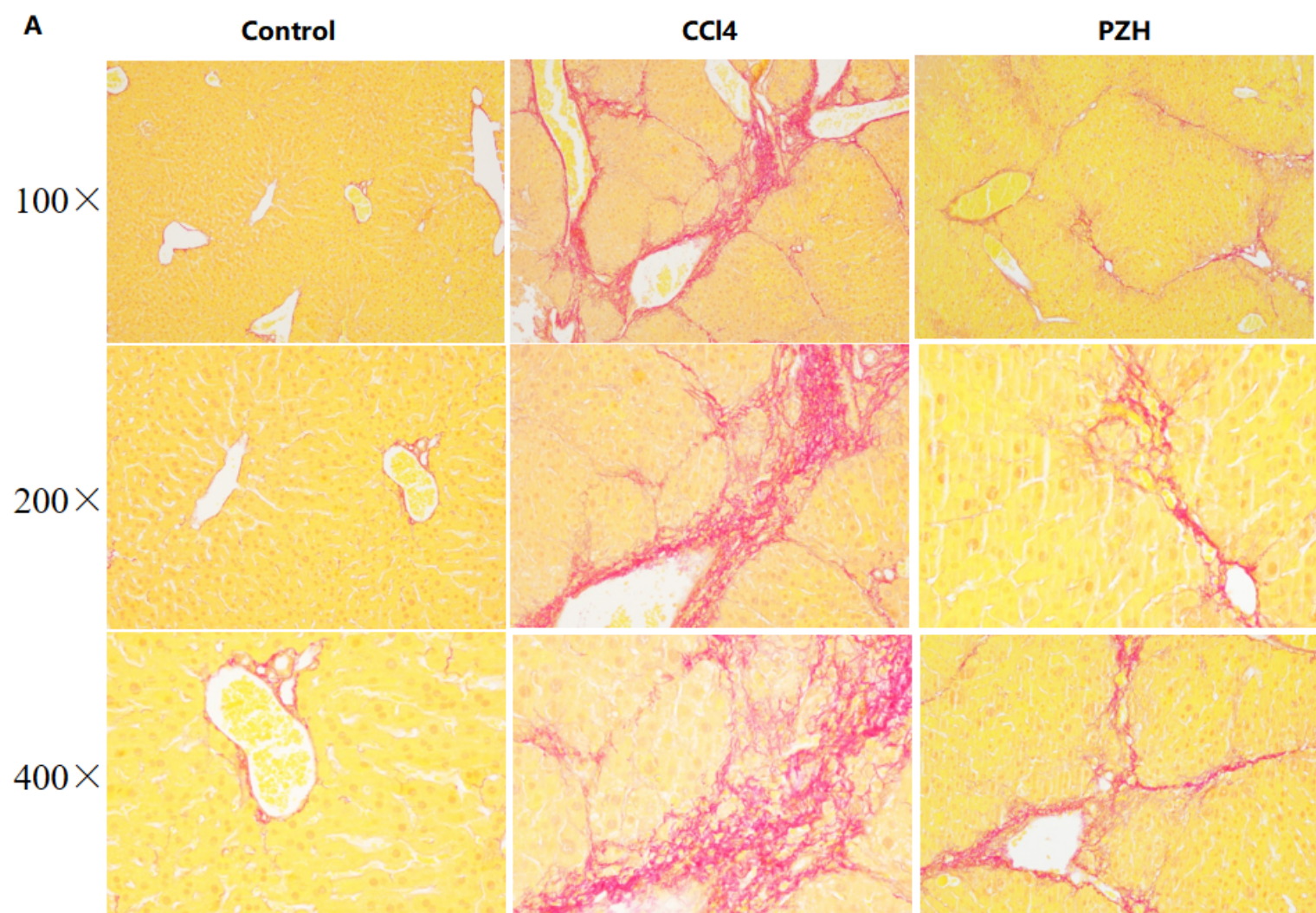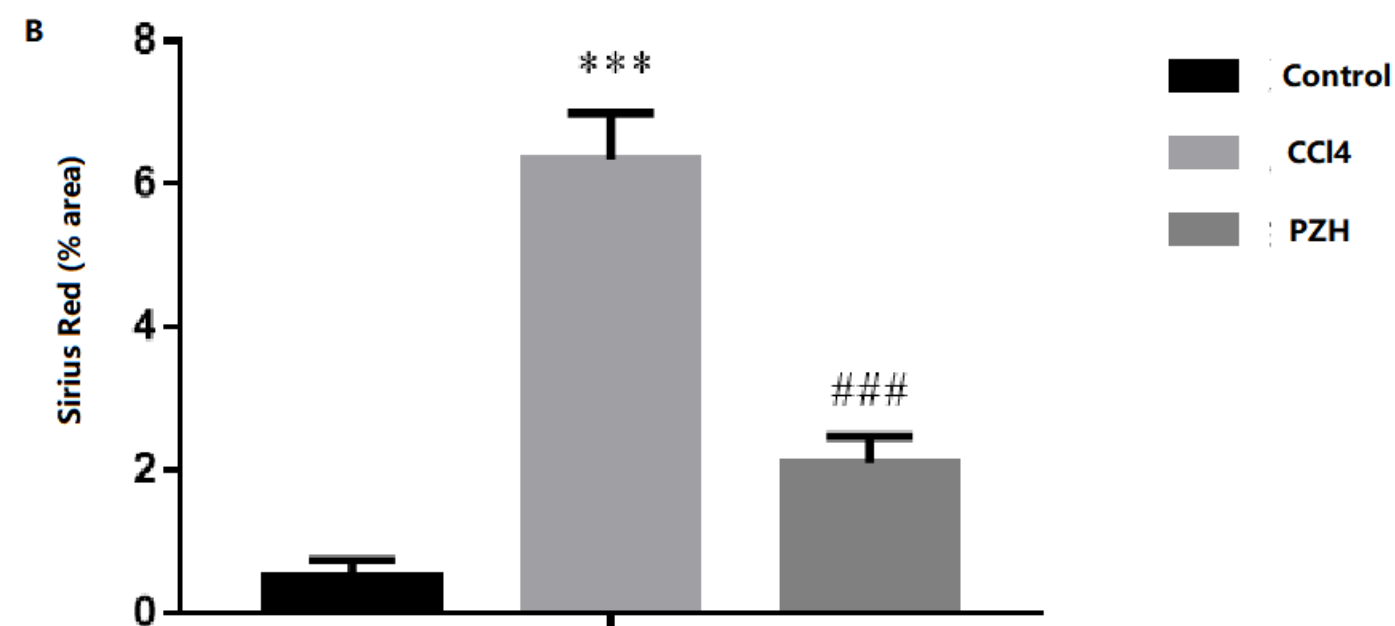

Supplement: Supplementary file 1 [file biomedicines-14-01550-s001.zip › Figure S1.pdf]
